# Effects of Maternal Nutrition on Female Offspring Weight Gain and Sexual Development

**DOI:** 10.3389/fgene.2021.737382

**Published:** 2021-11-23

**Authors:** Roberta Cavalcante Cracco, Fernando de Oliveira Bussiman, Guilherme Henrique Gebim Polizel, Édison Furlan, Nara Pontes Garcia, Diego Angelo Schmidt Poit, Guilherme Pugliesi, Miguel Henrique de Almeida Santana

**Affiliations:** ^1^ Department of Animal Science, College of Animal Science and Food Engineering – USP, Pirassununga, Brazil; ^2^ Departament of Veterinary Medicine, College of Animal Science and Food Engineering – USP, Pirassununga, Brazil; ^3^ Department of Animal Reproduction, College of Veterinary Medicine and Animal Science - USP, Pirassununga, Brazil

**Keywords:** beef heifer, fetal programming, Nellore, nutrigenetic, performance, reproduction

## Abstract

Maternal nutrition during pregnancy influences postnatal life of animals; nevertheless, few studies have investigated its effects on the productive performance and reproductive development of heifers. This study evaluated the performance, reproductive development, and correlation between reproduction × fat thickness and performance × ribeye area (REA) traits of heifers. We also performed an exploratory genomic association during the rearing period in heifers submitted to fetal programming. The study comprised 55 Nellore heifers born to dams exposed to one of the following nutritional planes: control, without protein-energy supplementation; PELT, protein-energy last trimester, protein-energy supplementation offered in the final third of pregnancy; and PEWG, protein-energy whole gestation, protein-energy supplementation upon pregnancy confirmation. Protein-energy supplementation occurred at the level of 0.3% live weight. After weaning, heifers were submitted to periodic evaluations of weight and body composition by ultrasonography. From 12 to 18 months, we evaluated the reproductive tract of heifers to monitor its development for sexual precocity and ovarian follicle population. The treatments had no effect (*p* > 0.05) on average daily gain; however, the weight of the animals showed a significant difference over time (*p* = 0.017). No differences were found between treatments for REA, backfat, and rump fat thickness, nor for puberty age, antral follicular count, and other traits related to reproductive tract development (*p* > 0.05). The correlation analysis between performance traits and REA showed high correlations (*r* > 0.37) between REA at weaning and year versus weight from weaning until yearling; however, no correlation was found for reproductive development traits versus fat thickness (*p* > 0.05). The exploratory genomic association study showed one single-nucleotide polymorphism (SNP) for each treatment on an intergenic region for control and PEWG, and the one for PELT on an intronic region of *RAPGEF1* gene. Maternal nutrition affected only the weight of the animals throughout the rearing period.

## 1 Introduction

The concept of fetal programming has emerged in recent decades and is used to explain metabolic and systemic changes due to events during fetal life (Barker, 1990). Nutritional changes, such as over- or under-nutrition, may occur with the mother and result in fetal programming, as reported by several studies (Wu et al., 2006; Long et al., 2009; Duarte et al., 2014). Systemic changes due to maternal nutrition include low birth weight, hormonal imbalances, and changes in organ development and functionality (Long et al., 2009; Micke et al., 2010a).

Moreover, studies show that fetal programming affects the reproductive system of both genders (Funston and Summers, 2013; Mossa et al., 2013; Mossa et al., 2017; Polizel et al., 2021). Other studies (Martin et al., 2007; Funston et al., 2010a) report the effects of nutrition during pregnancy on sexual precocity and pregnancy rate in heifers as well as on the reproductive potential of heifers in ovarian follicular reserve, even without changing other phenotypic characteristics (Mossa et al., 2013). Puberty is one of the most important periods for heifers, since it directly affects their productive, reproductive, and economic efficiency (Monteiro et al., 2013). The production of precocious animals is desirable, mainly to reduce the use of resources.

In Brazil, dams commonly undergo nutritional restriction due to the dry season present in tropical and subtropical conditions, especially during the second and third trimesters of pregnancy; therefore, investigations of undernutrition effects on progenies are needed to seek viable alternatives to overcome nutrient restriction during dry periods. In addition, few studies have investigated the effects of fetal programming on the performance of heifers in the rearing phase (Micke et al., 2010a; Long et al., 2012; Noya et al., 2019; Long et al., 2021).

The post-weaning period is crucial for the reproductive development of heifers, since the animals need to reach adequate body weight to attain puberty rapidly and then become replacement heifers or ready for finishing and slaughter. This study assessed the performance, reproductive development, and correlation between traits of heifers. We also performed an exploratory genomic association study during the rearing period in heifers submitted to different planes of maternal nutrition, with the objective of showing possible genotype–environment interactions, evaluating how individual genetic variants respond to nutritional stimuli. Therefore, our hypothesis is that different prenatal supplementation strategies influence weight gain and reproductive traits and that genetic variants influence the nutritional response in female offspring of Nellore dams.

## 2 Materials and Methods

### 2.1 Experimental Design

The study comprised 126 Nellore dams, which were fixed-time artificially inseminated (FTAI) with semen of four bulls with known genetic value, representing the majority of national Nellore animals. After confirmation of pregnancy at 30 days after FTAI, the animals were separated into three treatments: control, without protein-energy supplementation; PELT, protein-energy last trimester, protein-energy supplementation in the final third of pregnancy; and PEWG, protein-energy whole gestation, protein-energy supplementation upon pregnancy confirmation. The 126 animals were homogenized in the groups based on age (3–8 years), parity, body weight, and body condition score (Table 1), in order to make the groups as homogeneous as possible. Animals were allocated to pasture paddocks of *Brachiaria brizantha* cv. Marandu with access to the supplement (0.03% live weight for control and 0.3% live weight for PELT and PEWG) (Table 2) and water *ad libitum*. More details can be found on Polizel et al. (2021).

**TABLE 1:** Weight and BCS of dams on the beginning and end of gestation.

Traits	Time		Control	PELT	PEWG	*p*-Value
**Weight (kg)**	Initial		457 ± 9	453 ± 12	439 ± 16	0.96
		Min =	385	324	349	
		Max =	524	542	602	
	Pre-delivery		501 ± 10	523 ± 13	521 ± 18	0.20
		Min =	410	380	428	
		Max =	575	620	692	
	Postpartum		501 ± 10	502 ± 13	495 ± 16	0.91
		Min =	429	350	398	
		Max =	582	604	650	
**BCS**	Initial		4.5 ± 0.1	4.6 ± 0.1	4.4 ± 0.1	0.43
		Min =	4	3	3	
		Max =	5	5	5	
	Pre-delivery		5.4 ± 0.2	5.6 ± 0.2	5.5 ± 0.3	0.55
		Min =	4	4	4	
		Max =	7	7	7	
**Age (years)**			4.7 ± 0.3	4.5 ± 0.3	4.1 ± 0.3	0.48
		Min =	2.3	2.4	2.2
		Max =	6.2	7.2	6.2
**Parity**		Primiparous	17%	17%	19%	
		Multiparous	83%	83%	81%	

Note. The data are expressed as means of the characteristics ± standard error of the mean.

BCS, body condition score; Control, without protein-energy supplementation; PELT, protein-energy last trimester (0.3% BW protein-energy supplementation in the final third of pregnancy); PEWG, protein-energy whole gestation (0.3% BW protein-energy supplementation upon pregnancy confirmation).

**TABLE 2:** Ingredients and nutrients content of the dams supplement.

Ingredients	Mineral supplement	Energetic-protein supplement
**Corn (%)**	35	60
**Soybean meal (%)**	—	30
**Dicalcium phosphate (%)**	10	—
**Urea 45% (%)**	—	2.5
**Salt (%)**	30	5
**Minerthal 160 MD (%)** ^**a**^	25	2.5
**Total digestible nutrients (%)**	26.76	67.55
**Crude protein (%)**	2.79	24.78
**Non-protein nitrogen (%)**	—	7.03
**Acid detergent fiber (%)**	1.25	4.76
**Neutral detergent fiber (%)**	4.29	11.24
**Fat (%)**	1.26	2.61
**Calcium (g/kg)**	74.11	6.2
**Phosphate (g/kg)**	59.38	7.24

a Mineral pre-mix composition (guarantee levels per 25 kg): calcium, 200–300 g; cobalt, 160 mg; copper, 2,700 mg; sulfur, 60 g; fluorine, 1,600 mg; phosphor, 160 g; iodine, 135 mg; manganese, 2,700 mg; selenium, 80 mg; zinc, 8,100 mg; sodium monensin, 4,000 mg.

After calving, protein-energy supplementation ceased, and all animals remained together until weaning (average 220 days old), regardless of the treatment. The animals were subjected to the same sanitary, vaccination, and feeding protocols already implemented on the farm where the experiment was conducted. After weaning, the animals were divided by sex, regardless of treatment, and placed in separate pastures, where they remained throughout the breeding. The females remained on the pasture until the beginning of the reproductive season at 24 months. This trial comprised 55 heifers (control = 19, PELT = 22, and PEWG = 14), which were evaluated for reproductive development and performance regularly.

### 2.2 Reproductive Tract Assessment

The females were evaluated to determine the stage of reproductive development every 30 days from 12 months of age onward. Puberty was characterized based on the presence of corpus luteum (CL), and puberty age referred to the age in days of the animal of the first CL. A single specialized operator used an ultrasound machine equipped with a transrectal transducer (Mindray Z5 VET; Shenzhen Mindray Bio-Medical Electronics Co., Shenzhen, Guangdong, China) to qualify the CL presence. Antral follicle count (AFC) was performed to estimate the ovarian reserve at the same time that the presence of CL occurred, where a single operator visually enumerated the antral follicles ≥3.00 mm. Each ovary was investigated exhaustively throughout to standardize the count, identifying the positions of the antral follicles and capturing images of different sections of the organ. The size of each ovary was measured using its largest diameter, and the average size between the two ovaries was considered for each animal for statistical purposes. The thickness of the endometrial wall was also measured right after the corneal bifurcation during ultrasound, as described by Souza et al. (2011). The tonus and uterine sizes were also accessed through transrectal palpation, assigning scores (tonus = flaccid, minimal tonus or medium tonus; uterine size = infant, small, medium or developed) according to the perception of the evaluator and as proposed by Holm et al. (2009). For the statistical analysis, we used assessments at 12, 15, and 18 months, when evaluations of the reproductive tract ended.

### 2.3 Performance Evaluation

The performance of animals was evaluated in the periods of weaning, year (12 months), yearling (18 months), and 24 months, measuring weight and average daily gain (ADG). The ultrasound was used to measure ribeye area (REA), backfat thickness (BFT), and rump fat thickness (RFT). Weights were obtained regularly during the rearing period using an electronic scale from Coimma (Coimma Scales, Dracena, São Paulo State, Brazil) coupled to the trunk. The linear regression was performed using all collections between weaning and 24 months, totaling seven collections of weight, to obtain the ADG.

The body composition was evaluated by ultrasound using an Aloka SSD-500 ultrasound equipped with a 17-cm linear transducer at 3.5-MHz frequency (Aloka Co. Ltd., Wallingford, CT, USA). Vegetable oil was used as coupling to optimize the contact of the transducer with the skin of the animals. The REA and BFT were measured by images in sections of the *longissimus dorsi* muscle, between the 12th and 13th ribs, while the RFT was measured by positioning the transducer in the final portion of the ileum, between the junction of the biceps femoris and the middle gluteal muscle. The images were captured using the Lince software and later analyzed by a certified technician.

### 2.4 Nutrigenetic Evaluation

The DNA material was obtained from tail hair bulb; DNA extraction from the bulb of these hairs was performed by MICRO LAB ID STARlet^®^ automated robot (Hamilton Company, Reno, NV, USA) using the NucleoSpin^®^ 96 extraction kit (Macherey-Nagel, Düren, Germany). The 55 Nellore heifers were genotyped with the low-density panel GeneSeek^®^ Genomic Profiler Bos Indicus GGP Nellore LD BeadChip containing 35,339 markers; and before the imputation process, all single-nucleotide polymorphism (SNP) arrays had their maps converted to the new ARS UCD 1.2 reference genome. Imputation procedure was implemented using the FIMPUTE 2.2 software (Sargolzaei et al., 2014), and all genotypes were imputed to a panel containing 735,965 markers. A reference population with 2,502 sires and dams genotyped with the Illumina BovineHD BeadChip (Illumina Inc., San Diego, CA, USA) containing 777,962 markers was used. This population contains important and representative sires and dams within the Nellore breed, whose genetic material is widely used in breeding programs. Prior to imputation, samples were edited for call rate (<90%) for the genotyped and the reference populations. SNPs unassigned to any chromosome and those assigned to sexual chromosomes were removed from the dataset. After imputation, accuracy obtained was a mean (SD) of 0.93 (0.02), and genotypes presenting less than 0.90 of imputation accuracy were not considered in further analysis. The relationship degree between the target and reference population, was, on average (SD) of 0.08 (0.01).

We performed the genomic association analysis to understand the nutrigenetic effects of fetal programming, using the imputed SNP panel (35K) for reproductive characteristics (12, 15, and 18 months) and performance (weaning, year, yearling, and 24 months). Statistical information on models used can be found in *Nutrigenetics*. We used the packages SNPStats (Solé et al., 2006), gdata (Warnes et al., 2017), qvalue, data.table, ggplot2 (Wickham, 2011), and qqman (Turner, 2018). Concerning quality control (QC) for genomic data, all markers on sexual chromosome were removed from analysis, as well as markers with call rate <0.95 (0), with minor allele frequency <0.01 (209,831), *p*-value from the Hardy–Weinberg equilibrium <1 × 10^−10^ (26), and monomorphic (86,070). In addition, individuals with call rate <0.90 were also removed. Thus, the final genotypic data were left after QC with 55 individuals and 440,038 markers.

### 2.5 Statistical Analysis

#### 2.5.1 Phenotypes

All procedures were performed using the MIXED procedure of the statistical package SAS^®^ version 9.4 (SAS Institute Inc., Raleigh, NC, USA). From data obtained, residues were submitted to the Shapiro–Wilk test for normality implemented at UNIVARIATE procedure, where measurements that did not follow normality were transformed using log (i.e., ln(trait + 1)). The homocedasticity of residuals for principal effects was tested in the groups using Levene’s test. Then, the effects of treatments (control, PELT, and PEWG) on phenotypes were evaluated using the ANOVA, and the means were compared by the Tukey–Kramer test, with contrasts considered significant when *p* < 0.05 and trend when *p* < 0.10. The age of the animals, age of the dams, and sire were also considered in the linear model. Regarding the repeated measures, the same variables than linear model were considered, and time of data collection was also included in the ANOVA. The covariance structure for the residuals was tested for each variable and chosen based on the Bayesian information criteria (BICs). They were different for each variable, as follows: AFC used variance components (vc); weight used an autoregressive of first order (ar); REA used a compound symmetry structure for the residuals (cs); BFT used a heterogeneous compound symmetry (csh); and RFT used an autoregressive structure with moving average (arma).

For those analyses, the model was as follows:

$$y_{ijkl}=\mu +\beta_{1}Age_{m_{l}}+Sire_{i}+Treat_{j}+Time_{k}+{\left(Treat\times Time\right)}_{jk}+e_{ijkl}$$ (1)

where $y_{ijkl}$ is the observed variable from *l*th animal, daughter of *i*th sire, recorded on *j*th treatment at *k*th time of measurement (weaning, 12, 15, 18, and/or 24 months of age); μ is just a constant; $\beta_{1}$ is the regression coefficient of covariate mother’s age; $Age_{m_{l}}$ is the observed value for mother’s age of *l*th animal; $Sire_{i}$ is the fixed effect of *i*th sire; $Treat_{j}$ is the fixed effect of *j*th treatment; ${\text{Time}}_{\text{k}}$ is the fixed effect of *k*th time of measurement; ${\left(\text{Treat}\times \text{Time}\right)}_{\text{jk}}$ is the fixed interaction between treatment and time; and ${\mathrm{\&ExponentialE;}}_{ijkl}$ is the residual random term, which was assumed normally distributed with covariance structure as presented above. It must be noticed that when the analysis was performed within time, this effect (and also the treatment by time interaction) was removed from the model. Finally, for AFC, the weight at puberty was included as a covariate in the model.

The Kruskal–Wallis test was performed due to the scalar nature of the data collected for the characteristics of tonus and uterine sizes.

#### 2.5.2 Nutrigenetics

For nutrigenetics, two models were implemented through the “LM” function in R to correct the phenotype for the fixed effects, as follows:

$$y_{ijk}=\mu +\beta_{1}Age_{m_{k}}+Sire_{i}+Treat_{j}+\varepsilon_{ijk}$$ (2)

$$y_{ijk}=\mu +\beta_{1}Age_{m_{k}}+\beta_{2}Weight_{k}+Sire_{i}+Treat_{j}+\varepsilon_{ijk}$$ (3)

where $y_{ijk}$ is the observed phenotype of *k*th animal, daughter of *i*th sire, on *j*th treatment; μ is just a constant; $\beta_{1}$ is the regression coefficient of covariate mother’s age; $\beta_{2}$ is the regression coefficient of covariate weight at puberty (only for age at puberty); $Age_{m_{k}}$ is the observed value for mother’s age of *k*th animal; $Weight_{k}$ is the observed puberty weight of *k*th animal; $Sire_{i}$ is the fixed effect of *i*th sire; $Treat_{j}$ is the fixed effect of *j*th treatment; and $\varepsilon_{ijk}$ are random residual terms. The AFC observed values were transformed on log scale as: ln(AFC+1) and when the analysis was performed within each treatment, the treatment effect was not included in the model.

Under matrix notation, the models can be written as follows:

y=Xβ+ε (4)

where y is the phenotype vector; X is the incidence matrix for the fixed effects; β is the vector of solutions for the fixed effects; and ε is the vector of residual random terms. It was assumed that E[y]=Xβ ; $\varepsilon ∼N\left(0,I\sigma_{\varepsilon }^{2}\right)$ ; and thus $Var\left(y\right)=Var\left(\varepsilon \right)=I\sigma_{\varepsilon }^{2}$ . Under our assumptions, the residual can be re-written as (Searle, 1997) follows: ε=Zu+e , where Z is the incidence matrix for the animal additive effect; u is the solution vector for animal additive effect; and &ExponentialE; is the vector of true residuals.

After the solutions for β were obtained, we used ε as pseudo phenotypes for a genome-wide association study (GWAS) through the approach SNP by SNP; that is, each marker was fitted once. The adjusted phenotypes can be calculated as $\hat{\varepsilon }=y-X\hat{\beta }$ , where $\hat{\beta }$ is the empirical BLUE for the fixed effects. Thus, the GWAS was performed by the following model:

$$\hat{\varepsilon }=\beta_{0}+\beta_{1}PC1_{k}+\beta_{2}PC2_{k}+\beta_{3}SNP_{ki}+e_{i}=X\theta +e$$ (5)

where $\hat{\varepsilon }$ was the same as above; $\beta_{0}$ is the intercept; $\beta_{1}$ and $\beta_{2}$ are regression coefficients of the first and second principal components (PCs) from the genomic relationship matrix, respectively (VanRaden, 2008); $\mathrm{PC}1_{k}$ is the observed value of the first PC on *k*th animal; $\mathrm{PC}2_{k}$ is the observed value of the second PC on *k*th animal; $\beta_{3}$ is the regression coefficient of the marker effect (SNP effect); $SNP_{ki}$ is the scaled genomic content of *k*th animal on *i*th marker; ${\mathrm{\&ExponentialE;}}_{i}$ is the residual; θ is the vector of solutions for $\beta_{0}$ , $\beta_{1}$ , $\beta_{2}$ , and $\beta_{3}$ ; and &ExponentialE; is the vector of residual terms, which was assumed $\mathrm{\&ExponentialE;}∼N\left(0,I\sigma_{\mathrm{\&ExponentialE;}}^{2}\right)$ . For the GWAS, we also used the “LM” function within a loop for i from 1 to the total number of markers (440,038). After estimation of marker *p*-values, they were corrected for multiple testing by Bonferroni correction; i.e., the threshold for significance was set to 0.05/440,038.

#### 2.5.3 Correlation Between Performance, Body, and Reproductive Characteristics

Pearson’s correlation analysis was performed using the LM function of the statistical environment R to elucidate the relationship between the variables of weight, ADG, REA, BFT, RFT, age at puberty, AFC, ovary size, and endometrium thickness.

## 3 Results

On repeated measures, the interaction between time and treatment was not statistically significant for all studied traits (*p* > 0.05), but time was significant (*p* < 0.05) in all of them.

### 3.1 Weight and Average Daily Gain/Performance at Rearing Phase

The heifer’s weight in the three treatments was similar in all periods evaluated (*p* > 0.05); however, when an analysis was carried out over time, a significant difference occurred among the treatments (*p* = 0.017), where control was heavier and differed from the others. The ADG of the period showed no statistical difference between treatments (*p* > 0.05) with homogeneous weight gain (Table 3).

**TABLE 3:** Performance traits of Nellore heifers submitted to fetal programming.

Traits	Age	Control^A^ ^a^	PELT^B^ ^a^	PEWG^B^ ^a^	*p*-Value^b^	*p*-Value^c^
**Weight (kg)**	Weaning	216.4 ± 4.4	210.1 ± 4.7	208.3 ± 5.4	0.78	0.017
	Year	271.2 ± 4.1	263.9 ± 5.1	257.5 ± 5.4	0.37	
	Yearling	398.5 ± 5.5	384.9 ± 7.5	384.2 ± 7.3	0.39	
	24 months	428.7 ± 4.5	410.0 ± 6.6	410.7 ± 7.2	0.13	
**Ribeye area (cm^2^)**	Weaning	42.7 ± 1.2	43.5 ± 1.1	43.3 ± 1.5	0.92	0.929
	Year	55.7 ± 1.7	57.5 ± 1.1	56.3 ± 1.5	0.63	
	Yearling	73.5 ± 1.2	73.30 ± 1.5	75.0 ± 1.7	0.46	
	24 months	70.8 ± 1.1	70.8 ± 1.1	70.8 ± 1.5	0.83	
**Backfat thickness (mm)**	Weaning	2.85 ± 0.4	3.28 ± 0.4	2.72 ± 0.4	0.55	0.115
	Year	1.91 ± 0.3	1.94 ± 0.4	1.15 ± 0.4	0.19	
	Yearling	6.52 ± 0.6	7.41 ± 0.5	6.55 ± 0.6	0.27	
	24 months	5.70 ± 0.7	6.27 ± 0.5	6.20 ± 0.6	0.87	
**Rump fat thickness (mm)** ^**d**^	Weaning	4.90 ± 0.4	4.46 ± 0.4	4.35 ± 0.3	0.53	0.373
	Year	3.69 ± 0.4	3.36 ± 0.4	3.07 ± 0.5	0.51	
	Yearling	10.1 ± 0.7	10.2 ± 0.5	10.3 ± 0.7	0.95	
	24 months	8.81 ± 0.7	8.25 ± 0.5	8.99 ± 0.7	0.72	

Note. The data are expressed as means of the characteristics ± standard error of the mean.

Control, without protein-energy supplementation; PELT, protein-energy last trimester (0.3% BW protein-energy supplementation in the final third of pregnancy); PEWG, protein-energy whole gestation (0.3% BW protein-energy supplementation upon pregnancy confirmation).

a Refers to contrasts on weight characteristic.

b *p*-Value between groups on the same age.

c *p*-Value on repeated measures over time.

d Sire effect found on repeated measures over time (*p* < 0.05).

### 3.2 Fat Thickness and Ribeye Area

The body traits measured by ultrasound had no differences in the periods (*p* > 0.05), not even when the analysis was performed for repeated measurements over time, showing homogeneous fat and muscle deposition in these locations. However, in the RFT measurements, a difference related to a sire effect was found (*p* < 0.01) (Table 3).

### 3.3 Reproduction Traits

Puberty age and AFC of the treatments showed no significant differences between periods or over time (*p* > 0.05). There was no significant difference for ovary size and endometrial thickness in the periods and in the repeated measurements over time. However, ovary size had a significant difference for the age of animals (12, 15, and 18 months) (*p* < 0.05). The uterine size and tonus classificatory variables also displayed no differences between treatments (*p* > 0.05) (Table 4).

**TABLE 4:** Maternal nutritional effect on reproductive traits of Nellore offspring heifers.

Traits	Age	Control	PELT	PEWG	*p*-Value^a^	*p*-Value^b^
**Ovary size**	15 months	22.7 ± 0.6	22.0 ± 0.6	21.0 ± 0.6	0.32	0.37
	18 months	23.6 ± 0.6	24.6 ± 0.5	23.8 ± 0.5	0.34	
**Endometrium thickness**	15 months	6.2 ± 0.1	6.2 ± 0.2	6.2 ± 0.1	0.85	0.53
	18 months	5.6 ± 0.1	5.6 ± 0.1	5.3 ± 0.1	0.33	
**AFC**	15 months	15.1 ± 1.4	16.4 ± 1.0	17.1 ± 1.4	0.18	0.31
	18 months	15.6 ± 0.1	16.0 ± 1.2	16.4 ± 1.9	0.92	
**Age at Puberty**		475.76	474.94	475.33	0.87	

Note. The data are expressed as means of the characteristics ± standard error of the mean.

Control, without protein-energy supplementation; PELT, protein-energy last trimester; PEWG, protein-energy whole gestation; AFC, antral follicular count.

a *p*-Value between groups on the same age.

b *p*-Value on repeated measures over time.

### 3.4 Phenotypic Correlations

The correlation analysis between performance characteristics and REA showed a positive high correlation in weight at weaning vs. REA at weaning (*r* = 0.63), weight at year vs. REA at weaning (*r* = 0.55), weight at yearling vs. REA at weaning (*r* = 0.45), weight at weaning vs. REA at year (*r* = 0.41), weight at year vs. REA at year (0.53), weight at yearling vs. REA at year (*r* = 0.49), and weight at 24 months vs. REA at year (*r* = 0.37; *p* < 0.01). A positive moderate correlation was shown between weaning weight vs. REA at yearling (*r* = 0.30), weight at 24 months vs. REA at weaning (*r* = 0.28), weight at 24 months vs. REA at 24 months (*r* = 0.26), and ADG vs. REA at 24 months (*r* = 0.30; *p* < 0.05) (Table 5). When relating reproduction characteristics and fat thickness, no significant correlations were found between puberty ages, AFC and BFT, and RFT for the periods analyzed (*p* > 0.05) (Table 6).

**TABLE 5:** Pearson’s correlation between performance traits and ribeye area (REA).

Performance vs. REA	REAWE	REA12	REA18	REA24
**WWE**	0.63**	0.41**	0.30*	0.02
**W12**	0.55**	0.53**	0.38	0.17
**W18**	0.45**	0.49**	0.36	0.19
**W24**	0.28*	0.37**	0.23	0.26*
**ADG**	−0.02	0.09	0.19	0.30*

Note. WE, weaning; 12, year (12 months); 18, yearling (18 months); 24–24 months.

*p-Value < 0.05.

**p-Value < 0.01.

**TABLE 6:** Pearson’s correlation between reproductive traits and fat thickness.

Reproduction vs. fat thickness	BFTWE	BFT12	BFT15	BFT18	BFT24	RFTWE	RFT12	RFT15	RFT18	RFT24
**Age at puberty**	−0.05	−0.03	0.06	−0.05	−0.01	−0.12	0.00	0.13	0.06	−0.02
**AFC12**	−0.19	−0.2	−0.1	−0.07	−0.16	−0.08	−0.18	−0.03	−0.12	−0.2
**AFC15**	−0.02	−0.21	0.01	0.05	−0.08	0.04	0.02	0.11	0.19	0.07
**AFC18**	−0.08	−0.2	0.02	0.05	−0.15	−0.02	0.02	0.02	−0.05	−0.15

Note. WE, weaning; 12, year (12 months); 15–15 months; 18, yearling (18 months); 24–24 months.

*p-Value < 0.05.

**p-Value < 0.01.

### 3.5 Exploratory Genomic Association Study

**FIGURE 1:**
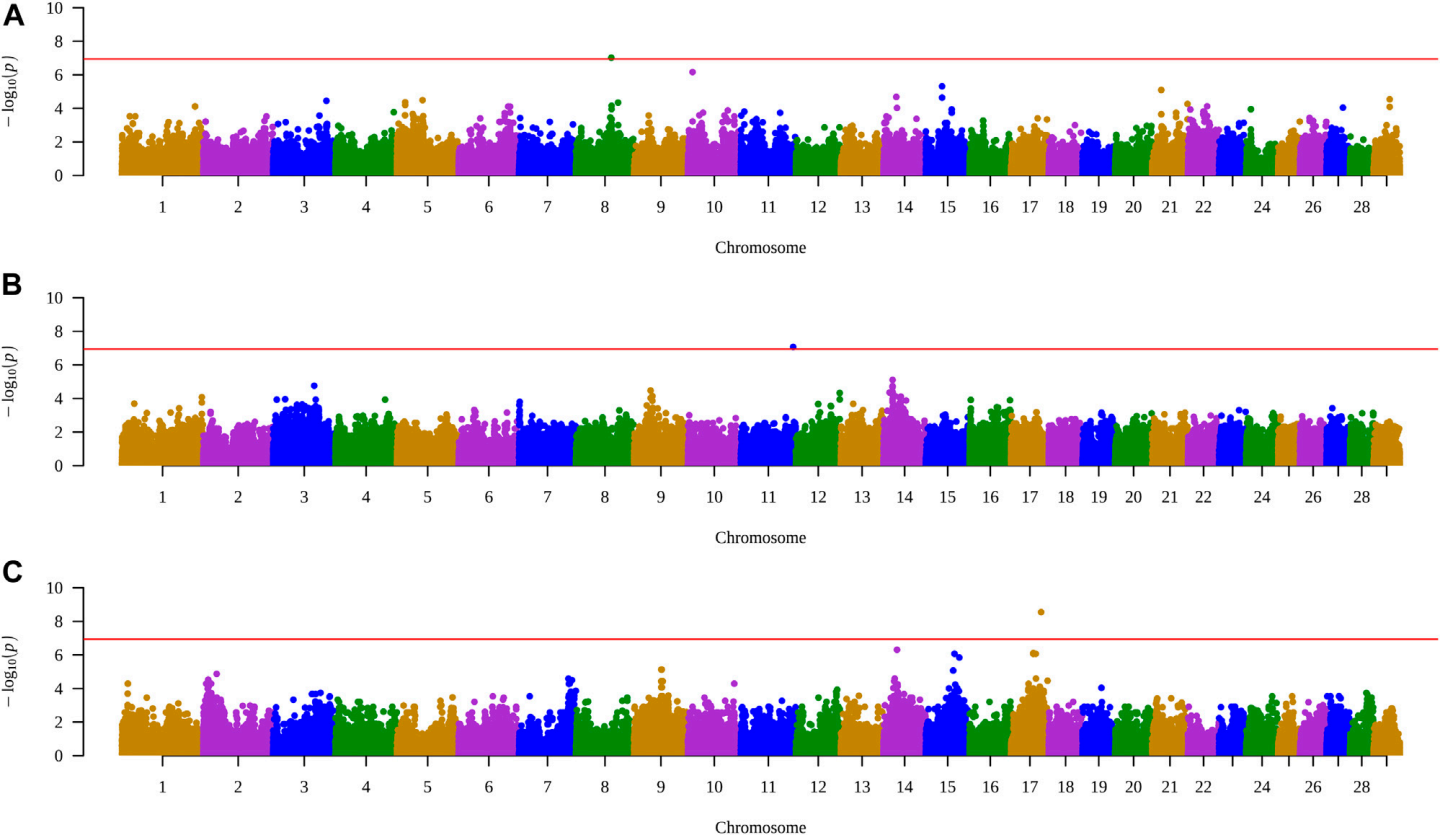
Manhattan plots of traits with SNPs highlighted by the exploratory genomic association analysis in each treatment. **(A)** AFC at yearling in control group. **(B)** Weight at yearling in PELT group. **(C)** BFT at year in PEWG group. SNPs, single-nucleotide polymorphisms; AFC, antral follicle count; PELT, protein-energy last trimester; BFT, backfat thickness; PEWG, protein-energy whole gestation.

**FIGURE 2:**
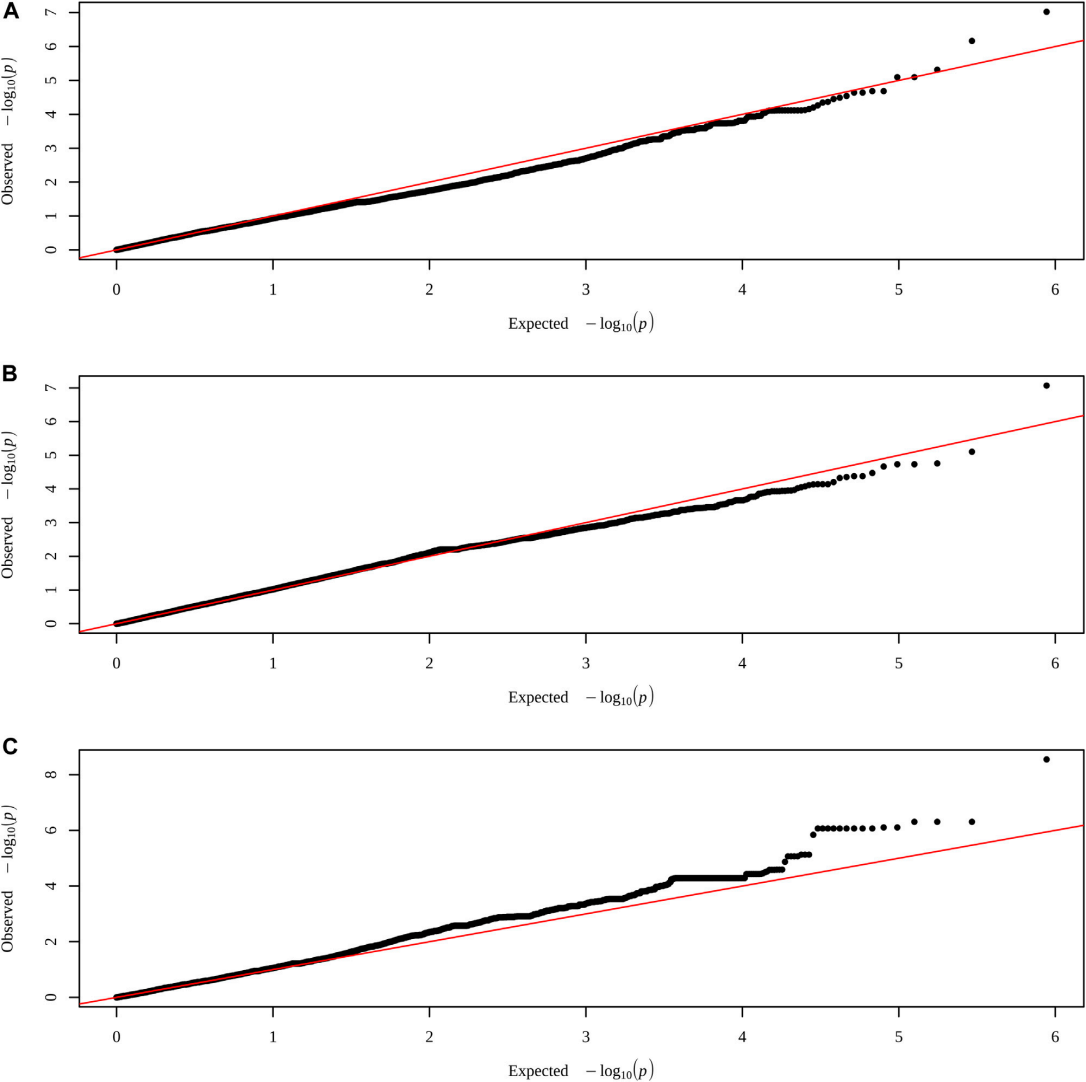
QQ plots of traits with SNPs highlighted by the exploratory genomic association analysis in each treatment. **(A)** AFC at yearling in control group. **(B)** Weight at yearling in PELT group. **(C)** BFT at year in PEWG group. SNPs, single-nucleotide polymorphisms; AFC, antral follicle count; PELT, protein-energy last trimester; BFT, backfat thickness; PEWG, protein-energy whole gestation.

When all animals were analyzed, no SNP had significance for any of the characteristics. However, when performing the analysis within each group, a significant SNP was identified for each treatment, with control in the trait AFC at yearling, PELT for weight at yearling, and for PEWG, and BFT at year (Figures 1, 2). The SNPs of the control and PEWG treatments are in the intergenic region, and the SNP of the PELT treatment is an intron variant of *RAPGEF1* gene (Table 7). For the SNPs in the intergenic region, we considered candidate genes within a window of 1 Mb around the marker. Only one gene, in AFC at yearling’s SNP (*GFRA2*), was as close as 100 kb from the marker. The data for traits that had significant SNPs can be found in the Supplementary Table S1.

**TABLE 7:** SNPs highlighted by the exploratory genomic association analysis.

Characteristic	Treatment	SNP	−log_10_ *p*	Gene associated
AFC at yearling	Control	rs135063035 Location 8:68964611	9.51 × 10^−8^	*GFRA2* (d)
				*XPO7* (d)
				*DOK2* (d)
				*NPM2* (d)
				*FGF17* (d)
				*DMTN* (d)
				*HR* (d)
				*FAM160B2* (d)
				*NUDT18* (d)
Weight at yearling	PELT	rs110561890 Location 11:101799978	8.52 × 10^−8^	*RAPGEF1*
BFT at year	PEWG	rs137051110 Location 17:58480895	2.82 × 10^−9^	*SPRING1/C12ORF49* (u)
				*RNFT2* (u)
				*FBXW8* (u)
				*ENSBTAG00000037415* (u)
				*ENSBTAG00000053074* (u)
				*MED13L* (d)

Note. (d) or (u) refer to whether the gene is upstream (u) or downstream (d) of their related SNP.

SNP, single-nucleotide polymorphism; AFC, antral follicle count; PELT, protein-energy last trimester; BFT, backfat thickness; PEWG, protein-energy whole gestation.

## 4 Discussion

This study investigated the potential effects of protein and energy supplementation on dams during the entire gestation and in the final third of gestation as well as on cows that did not receive supplementation, regarding performance in the rearing season and reproductive tract development. To date, few studies have related the rearing phase of beef heifers to fetal programming. Here, we did not find differences in sexual development; however, there were differences in body weight throughout the rearing period, showing the contribution of this study to this research field.

Some studies have shown that energy restriction during fetal life can negatively affect growth and performance in postnatal life, including body composition (Daniel et al., 2007; Du et al., 2010); nevertheless, few studies report its effects on females, especially on *Bos indicus* heifers. Micke et al. (2010b) reported that supplemented heifers were heavier than nonsupplemented ones. However, Long et al. (2012) found no difference between treatments. That study used heifers from dams with or without nutritional restriction during the final third of gestation and identified no differences between the groups for weight, ADG, and REA in the analyzed periods. In the same study, the authors reported that the progeny of dams that underwent undernutrition in the final third of gestation had greater deposition of internal fat, which may help explain why the control group was heavier than the others over time. Daniel et al. (2007) observed that ewes that were nutrient-restricted during pregnancy showed no effect of maternal nutrition on the deposition rate of muscle and fat in the progeny, corroborating our results of REA, BFT, and RFT. Reis et al. (2015) studied calves that underwent or not creep-feeding during the nursing period and reported no differences for BFT between treatments. The contradictory results of several studies reinforce the need to further investigate the mechanisms of fetal programming.

Puberty in heifers is defined as the age when the animal experiences its first ovulation accompanied by visual signs of estrus and normal luteal function (Moran et al., 1989), an important characteristic, as pregnancy success during the breeding season is associated with the number of heifers that reached puberty before the season (Short and Bellows, 1971). Weight is the most important factor for puberty onset, since puberty is achieved when the animal is between 55% and 60% of its mature body weight, regardless of the breed (Freetly et al., 2011). Studies on fetal programming show the effects of supplementation during pregnancy on puberty age in heifers (Guzmán et al., 2006; Funston et al., 2010b; Harvey et al., 2021). Other investigations show no effects (Cushman et al., 2014; Gunn et al., 2015; Nepomuceno et al., 2017), corroborating the lack of difference between treatments. Previous studies have indicated that maternal nutrition during pregnancy can interact with nutrition in early postnatal life to determine the puberty age in heifers (Cardoso et al., 2020). Furthermore, although postnatal nutrition has more significant effects than maternal nutrition, heifers from mothers that underwent nutritional restriction were more sensitive to the negative effects of limited postnatal growth (O’Neil et al., 2019). Therefore, it is justifiable that the heifers used in our study do not show differences of puberty ages when they start receiving the same environmental conditions, regardless of maternal treatment groups, and did not undergo nutritional restrictions that could limit their postnatal growth in their first months of life, since the animals were born in the rainy season. Moreover, although there was no difference between groups, the mean age at puberty (16 months) was earlier than the Nellore mean, between 22 and 36 months (Nogueira, 2004); also the body weight of the animals in this study was greater than literature reports for Nellore females (Boligon and Albuquerque, 2011). A point to be reinforced is that up to 24 months, animals received an excellent nutritional management, which contributed to body development in general. However, the effect under more restricted conditions can produce different results and needs to be evaluated in future research.

The AFC is an important marker of ovarian follicle reserve (Mossa et al., 2012) and thus of the animal reproductive efficiency. Studies associate maternal malnutrition during pregnancy to a low AFC (Mossa et al., 2013). The formation of primordial cells, precursors of follicles, begins between the 90th and 140th days of fetal life in cattle (Yang and Fortune, 2008); therefore, in our study, the treatments without supplementation in this period (second third of gestation; control and PELT) may have suffered the undernutrition effects due to the dry season. Nevertheless, undernutrition was possibly not severe enough. In our study, we used cows, which possibly affected these results. On the other hand, Mossa et al. (2013) used heifers, which are still growing in addition to having to spend energy for their maintenance and gestation, as hypothesized by Cushman et al. (2014).

One way to assess pubertal status indirectly is through palpation of the reproductive tract (Holm et al., 2009). Andersen et al. (1991) developed a standard method for the reproductive tract score, a tool to access the animal proximity to puberty by the uterine size and tonus sizes of ovary and structures in the organ. In our study, we used these characteristics to investigate effects of maternal nutrition in the gestational period on the development of the reproductive tract of heifers. However, with the absence of statistical differences in these characteristics and in the puberty ages between treatments, we can suggest that maternal nutrition did not affect the offspring’s reproductive tract development.

The correlation between the phenotypes shows the association degree between them, or a measurement of the joint variation degree. According to the results in Table 3, REA at weaning and at 1 year of age showed a positive correlation with weights between weaning and 24 months. This correlation can be explained by animal growth, since heavier heifers have greater REA (Minick et al., 2002). At 18 months, the correlation between REA and weight is practically nonexistent, possibly because the animals entered puberty and decreased muscle deposition, switching it for fat deposition (Owens et al., 1995). Since the phenotypes were corrected for the fixed effects before calculating the correlation, part of this coefficient takes into account the genetic value of the animals. Therefore, despite the small size of the database, it is still plausible to assume that part of this coefficient is a good approximation for the genetic correlation. Studies show a correlation between weight and REA (Lamb et al., 1990; Splan et al., 1998) and between ADG and REA (Mahmood et al., 2016). The correlation between reproductive tract and fat thicknesses showed a possible association between the characteristics, since RFT was already related to the reproductive tract and became more significant, as the heifers became more mature (Minick et al., 2002). Another factor with a great effect on the expected result is the relationship between leptin and puberty onset. Leptin is a hormone produced by adipose tissue and has a direct action on the hypothalamus–pituitary–ovary axis, causing an increase in peaks of GnRH (Zambrano et al., 2006; Duittoz et al., 2016; Perry, 2016).

In addition, we conducted an exploratory study on the genomic association that resulted in a significant SNP for each treatment. SNPs related to control and PEWG treatments are located in the intergenic regions, and the affected genes are often difficult to determine (Brodie et al., 2016). However, some genes found near the intergenic regions of SNPs have functions related to the characteristic in which it was identified. The SNP related to the control treatment is linked to the characteristic AFC at yearling. *NPM2* gene relates to the function of the ovarian and reproductive tract and encodes an oocyte-specific nuclear protein, with great importance in early embryonic development (Lingenfelter et al., 2011). *FGF17* gene plays a role in the differentiation of granulosa cells (Machado et al., 2009) and also in hypogonadism (Miraoui et al., 2013). On the other hand, *GFRA2* gene, the only gene within 100 kb from the marker, has an important role in the differentiation of stem cells in the pituitary (Pradilla et al., 2021), and important organ related to reproductive development. Gene *XPO7* has been linked to ovarian cancer (Cáceres-Gorriti et al., 2014), and *DOK2* gene was related to fetal programming, having its gene expression reduced in offspring of animals that underwent uteroplacental insufficiency (Master et al., 2015).

Several genes related to lipid metabolism were found close to the SNP for BFT at year, which is related to the PEWG treatment. *SPRING1* gene, also known as *C12orf49*, is an important regulator of lipid metabolism homeostasis (Aregger et al., 2020; Girardi and Superti-Furga, 2020). Studies on characteristics of buffalo milk link *RNFT2* gene to the production of fat, proteins, and milk (Venturini et al., 2014; Du et al., 2019). *FBXW8* gene was associated with fetal programming in a study that analyzed intrauterine growth restriction (Gascoin-Lachambre et al., 2010). *MED13L* gene is associated with heart development in humans (Napoli et al., 2019). *RAPGEF1*, the significant gene for the PELT treatment, has been studied and found to be related to persistence of lactation (Do et al., 2017) and mastitis in dairy cows (Chen et al., 2015) also to the lipomatous myopathy disease in Piedmontese cattle (Peletto et al., 2017). Nevertheless, these results need to be considered with great parsimony, since the sample size used in the analysis is small, which can lead to false-positive results. Our work investigated possible genotype–environment interactions in animals submitted to fetal programming. This is an innovative study, since no studies evaluated animals under these conditions and phenotypes. Furthermore, the data presented will attain greater accuracy in future studies, as the fetal programming database increases its amount of information.

## 5 Conclusion

Protein-energy supplementation at different gestation periods in Nellore cows did not affect the reproductive tract development or body composition and ADG during the rearing period of their daughters. However, the treatment affected weight over time, and the animals of the control group were heavier. The exploratory genomic association study showed one SNP on an intergenic region for control and PEWG, and one for PELT in an intronic region of *RAPGEF1* gene. Our study provided insights into the effects of fetal programming on Nellore heifers, showing that protein-energy supplementation may not affect their sexual development. However, this field requires further studies once the results found in literature are still contradictory and research with exploratory GWASs in animals that have undergone fetal programming is still scarce.

## Data Availability

The raw data supporting the conclusion of this article will be made available by the authors, without undue reservation.
